# Association of SNP rs80659072 in the ZRS with polydactyly in Beijing You chickens

**DOI:** 10.1371/journal.pone.0185953

**Published:** 2017-10-09

**Authors:** Qin Chu, Zhixun Yan, Jian Zhang, Tahir Usman, Yao Zhang, Hui Liu, Haihong Wang, Ailian Geng, Huagui Liu

**Affiliations:** 1 Institute of Animal Husbandry and Veterinary Medicine, Beijing Academy of Agriculture and Forestry Sciences, Beijing, P. R. China; 2 College of Veterinary Science and Animal Husbandry, Garden Campus, Abdul Wali Khan University Mardan, Mardan, Pakistan; Universita degli Studi di Bologna, ITALY

## Abstract

The Beijing You chicken is a Chinese native breed with superior meat quality and a unique appearance. The G/T mutation of SNP rs80659072 in the *Shh* long-range regulator of GGA2 is highly associated with the polydactyly phenotype in some chicken breeds. In the present study, this SNP was genotyped using the TaqMan detection method, and its association with the number of toes was analyzed in a flock of 158 birds of the Beijing You population maintained at the Beijing Academy of Agriculture and Forestry Sciences. Furthermore, the skeletal structure of the digits was dissected and assembled in 113 birds. The findings revealed that the toes of Beijing You chickens were rich and more complex than expected. The plausible mutation rs80659072 in the zone of polarizing activity regulatory sequence (ZRS) in chickens was an essential but not sufficient condition for polydactyly and polyphalangy in Beijing You chickens. Several individuals shared the T allele but showed normal four-digit conformations. However, breeding trials demonstrated that the T allele could serve as a strong genetic marker for five-toe selection in Beijing You chickens.

## Introduction

**Fig 1:**
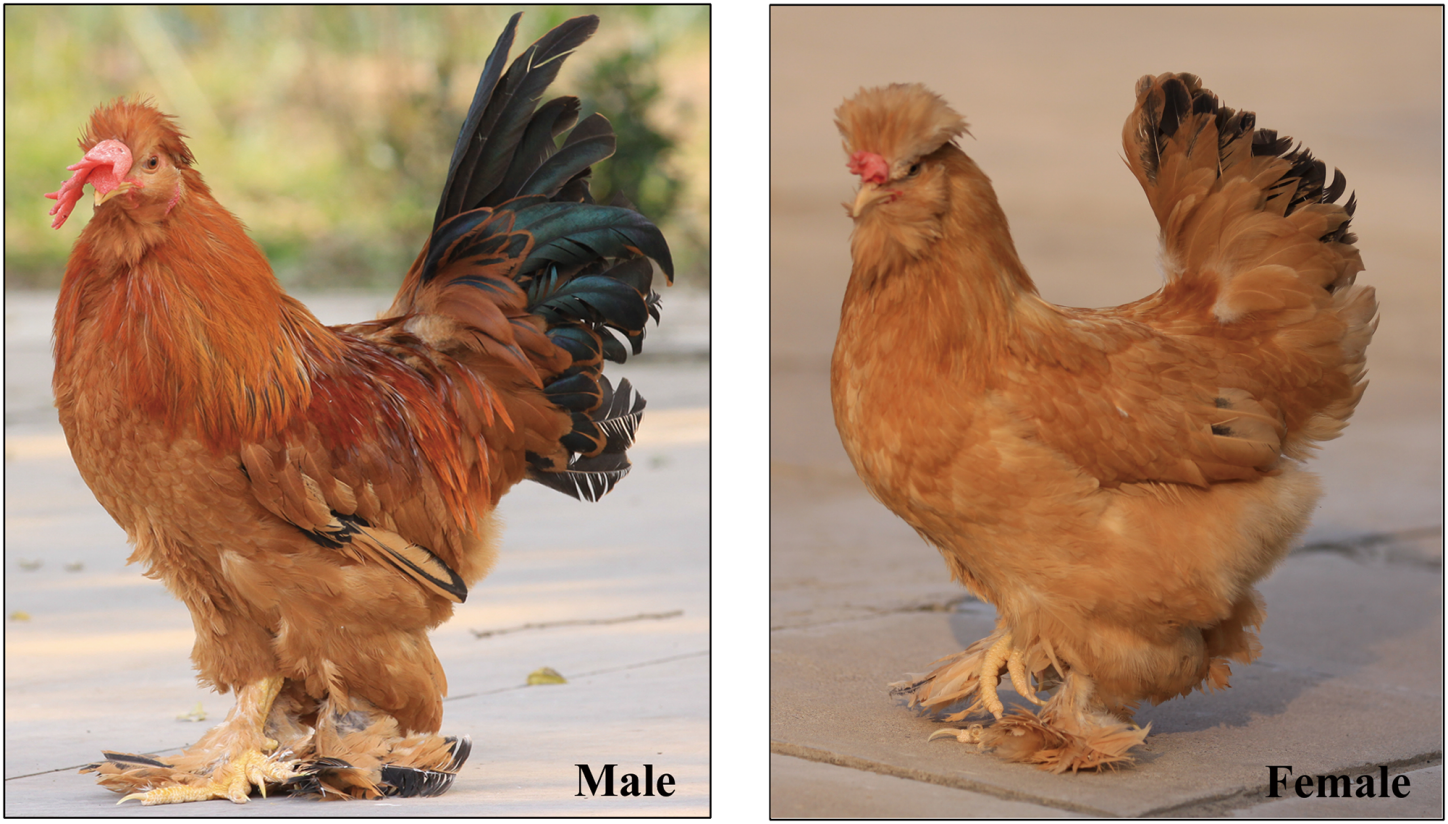
Pictures of male and female Beijing You chickens. The crest on the head, beard under the lower jaw, and feathers on both shanks are obvious.

The Beijing You chicken is an ancient native Chinese chicken breed that originated in the Qing Dynasty in Beijing, China. Beijing You chicken is known for its high-quality meat and eggs and unique appearance [1, 2], which includes a crest on the head, a beard under the lower jaw, and feathers on both shanks (Fig 1). Individuals of this breed may possess five or more toes on one or both feet (polydactyly), a characteristic also exhibited by the Houdan, Dorking and Silkie breeds [3, 4]. Polydactyly is a common congenital hereditary limb malformation that occurs in chickens and other vertebrates, including humans [5–7], pigs [8], dogs [9, 10], and cattle [11–13].

In China, native customers prefer chickens with unique appearance characteristics. Thus, breeders and producers focus not only on the growth rate and reproductive efficiency of chickens but also on their plumage color, body shape, comb shape, skin, shank color, and other qualities [14]. For Beijing You chickens, the number of toes could be considered a breed characteristic and an apparent marketable trait that may facilitate customer decision making. To achieve a population uniquely with five toes on both feet, we launched a three-year selection program by only keeping bilaterally five-toed birds since 2008 [15]. However, 12% of the chicken population was still found to possess four toes on both or one side. This phenomenon evoked us to decipher the genetic mechanism underlying this trait in Beijing You chickens.

The sonic hedgehog (Shh) signaling pathway is essential for regulating digit number and identity [16–19]. Mutations or genomic duplications in the zone of polarizing activity regulatory sequence (ZRS), a long-range limb-specific cis-regulator of *Shh* have been widely reported to cause preaxial polydactyly in humans [6, 7, 20–22]. Similarly, QTL mapping and GWA studies showed that the chicken polydactyly mutation was linked to the chromosome 2p region [3, 4, 23–25]. The G/T mutant of SNP rs80659072 (also called ss161109890) locating in the known 794-bp chicken ZRS region on GGA2p is responsible for the polydactyly phenotype in White Sultan [3] and Chinese indigenous chicken breeds, including Beijing You flocks [2, 4]. This genetic variant was documented to initiate ectopic *Shh* expression in the anterior leg by altering transcription factor binding that resulted in preaxial polydactyly [17]. However, in some polydactylous breeds, such as Salmon Faverolle, Mottled Houdan and Silver Gray Dorking, this phenotype was caused by different mutation events [3, 4].

Herein, the aim of the present study was to evaluate the association between SNP rs80659072 and the number and specific conformation of digits in a mass Beijing You chicken population maintained at the Beijing Academy of Agriculture and Forestry Sciences. The findings of the present study provide valuable information for marker-assisted selection for toe number conformation in Beijing You chickens.

## Materials and methods

### Ethics statement

This study was performed in accordance with the animal welfare guidelines of the Institutional Animal Care and Use Committee (IACUC) of the Institute of Animal Husbandry and Veterinary Medicine, Beijing Academy of Agriculture and Forestry Sciences (Permit number: 2015-YJ-03). All efforts were made to alleviate animal suffering.

### Animals and foot phenotypes

A flock of 158 birds, including 38 male and 120 female Beijing You chickens were randomly selected from a conservation population raised at the Beijing You Chicken Conservation Farm, Beijing Academy of Agriculture and Forestry Sciences. This breed has been conserved since 1972, with the original flocks collected from the folk [26]. The stemma equimultiple random select cross method has been adopted to propagate offspring, where the average inbreeding coefficient was 0.004 in the year 2016. The foot digit pattern was carefully recorded as 4/4 (four toes on both feet), 5/4 (five toes on the left foot and four toes on the right side), 4/5 (four toes on the left and five toes on the right side), 5/5 (five toes on both feet), and 6/- (six toes on one or both feet).

### TaqMan detection of the ZRS SNP

Trained veterinarians collected all blood samples via superficial venipuncture of the wing vein using EDTA-coated blood tubes following standard procedures. Genomic DNA was extracted from whole blood using the Blood DNA Kit DP304 (Tiagen, Beijing, China) according to the manufacturer’s instructions, and the concentration of DNA was measured using a NanoDrop 2000 spectrophotometer (Thermo Scientific, Wilmington, DE, USA). The DNA samples were stored at 4°C until further use.

Sequences surrounding the ZRS region were downloaded from the UCSC genome browser (http://genome.ucsc.edu). The primers and probes were designed using the Primer Express software, version 3.0 (Applied Biosystems, Foster City, CA, USA). The primers and fluorescence-labeled TaqMan MGB probes used in the present study are listed in Table 1.

**Table 1:** Information for the primers and probes used in the real-time PCR assay.

	Sequence (5’ to 3’)	Position^a^	Reporter (5’)	Quencher (3’)
**Primer**				
ZRS-FWD	5’- TCAGTGGCAAAAAACGAGCAAAAAT-3’	8467207–8467231		
ZRS-REV	5’- CACACAGAAATGAGTAGGAAGTCCAA -3’	8467272–8467299		
**Probe**				
ZRS-wt	5’-ATGCAATGAAAGCTC-3’	8467241–8467255	VIC	MGB-NFQ^b^
ZRS-mt	5’- CATGCAATTAAAGCTC-3’	8467240–8467255	FAM	MGB-NFQ

^a^ The position was based on the published chicken sequence (www.ensembl.org, galgal4).

^b^ MGB-NFQ = minor groove binder-nonfluorescent quencher.

PCR assays were performed in a total volume of 10 μL, which contained 10 ng of DNA template, 0.25 μL of 40X SNP Genotyping Assay Mix (the primer and probe concentrations were 36 and 8 μM, respectively), 5 μL of 2X TaqMan^®^ Genotyping Master Mix and 3.75 μL of double-distilled water. Real-time PCR was performed using the Bio-Rad iQ™5 real-time PCR detection system (Bio-Rad, Hercules, CA, USA) and the following program: 95°C for 10 min, followed by 40 cycles of 95°C for 15 s and 60°C for 1 min. The data were continuously collected during thermocycling at the annealing step. The samples were amplified, read and analyzed using the iQ^™^5 optical system software. Two controls (no template) in each 96-well plate were used for the assay quality control.

### PCR and sequencing

Variants of ZRS in GGA2 were screened using a DNA pooling strategy with primers described by Dorshorst et al. [3]. Using the 158 genomic DNAs, nine pools with a sample size less than 25 were constructed, including three for 4/4, three for 5/5, and one each for the other three phenotypes.

To verify the accuracy of the TaqMan method, three samples from each genotype were randomly selected and sequenced. The PCR assay was performed using a pair of primers designed using Primer3 online software (http://primer3.wi.mit.edu/). The sense primer sequence was 5’-ACATACCAAGAATGTGCATGTGC-3’, and the antisense primer sequence was 5’-TTTGAGGTAACTTCCTTGCTTAA-3’.

PCR amplification was conducted in a total volume of 20 μL, which contained 100 ng of DNA template, 1 μM of each primer, 200 μM dNTPs, 2.5 units of Taq DNA polymerase (Takara, Japan), 2 μL of 10X Buffer (Takara, Japan), and 1.5 mM MgCl_2_. PCR cycling was performed using the following program: 94°C for 5 min; followed by 30 cycles of 94°C for 30 s, 62°C for 30–40 s and 72°C for 30 s; and a final extension at 72°C for 7 min. The PCR products were detected using electrophoresis on a 2% agarose gel stained with ethidium bromide and then sent to the Beijing Tsingke Biotechnology Company (Beijing, China) for sequencing.

### Mating scheme

To observe the inheritance patterns, a total of 87 chickens with different foot phenotypes and ZRS SNP genotypes were selected from the aforementioned 158 birds. For each mating scheme, random mating was adopted to avoid inbreeding between sibs. Hatching eggs of breeder hens were collected from 45–50 week-old hens and stored at 16°C at 50 to 60% RH. Each collection time was 15 days long. Two batches of eggs were incubated and hatched under the same conditions, i.e. 38.0°C for the first 18 days and 37.6°C for the last three days. The RH was 65% in the first week, 56% from 8 to 19 days, and 75% for the last three days. The eggs were automatically turned every 2 hours until 19 days of incubation. At birth, the toe patterns on both feet of all chicks were recorded.

### Anatomical observation

Out of the 158 chickens, 116 with different phenotypes were electrically stunned (11 V, 11 mA, 10 s) and subsequently slaughtered, including 87 in the mating scheme and 29 having the other three kinds of phenotypes (4/5, 5/4 and 6/-). Total 232 feet were harvested, including 106 four-toed feet, 106 five-toed feet and 20 six-toed feet obtained from forty-four 4/4 birds, forty-three 5/5 birds, thirteen 5/4 birds, four 4/5 birds, eight 6/6 birds, three 6/5 birds and one 6/4 bird. After proper labeling, the feet were subsequently boiled in drinking water until the muscles and tendons could be easily removed from the bones. The tarsometatarsus, metatarsals, and phalanges from each chicken were then dissected and placed back together one by one according to their original order. Finally, these digit skeletons were posed in the correct manner and subsequently photographed (Canon EOS 5D Mark III, Japan).

### Data analyses

For many of the expected value from phenotype 5/4, 4/5 and 6/- were less than five, these classes were considered as a polydactylous group together with 5/5. Genotype frequency differences between four-toed and polydactylous chickens were assessed using a Chi-square test with the SAS software (Version 9.2).

## Results

### Association between digit phenotypes and SNP rs80659072 polymorphisms

Using the established TaqMan detection method, the genotypes of 158 flocks were determined. The sequencing results of different genotypes confirmed the accuracy of this technology, and no new mutations were observed.

The SNP detection results and digit patterns are shown in Table 2. The genotype distribution for the ZRS SNP rs80659072 was significantly different between different phenotypes (χ^2^ = 79.63, df = 2, and *P*<0.0001). Among all 158 chickens, 64 birds had the 4/4 digit pattern, of which 10 birds had TT genotypes, 14 birds had GT heterozygotes, and the other 40 individuals had the expected GG genotypes. All birds with more than four toes on one foot or both, including the 5/4, 4/5, 5/5 and 6/- digit patterns, had either the TT or GT genotype. None of the individuals with the GG genotype represented one or more supernumerary digits, suggesting that the T allele is an essential element for polydactyly or the extra digit condition in Beijing You chickens.

**Table 2:** Foot phenotypes and genotypes in Beijing You chickens.

Phenotype	Number of chickens	Genotype		
		TT	GT	GG
4/4	64	10	14	40
5/5	65	38	27	0
5/4	13	2	11	0
4/5	4	2	2	0
6/-	12	10	2	0
Total	158	62	56	40

However, possessing more than a T allele at the rs80659072 site was not always consistent with a toe number of more than four. In this population, 16.13% (10/62) and 25% (14/56) of the birds from the TT and GT groups exhibited a 4/4 digit pattern, respectively.

### Inheritance analysis

Chickens with different genotypes and phenotypes were mated to evaluate the inheritance patterns. The mating schemes were divided into several types as illustrated in Table 3.

**Table 3:** Mating results among different phenotypes and genotypes.

Genotype	Rooster:hen	Total progeny number	Digit phenotype of progeny				
			4/4	5/4	4/5	5/5	6/-
Symmetrically four-toed							
GG×GG	7:20	363	363	0	0	0	0
TT×TT	3:7	20	6	1	3	10	0
TT×GT	3:7	53	7	6	4	36	0
Symmetrically five-toed							
TT×TT	8:20	310	5	11	5	287	2
TT×GT	3:6	72	3	3	2	64	0
GT×GT	2:4	19	8	0	0	10	1

#### Mating between symmetrically four-toed birds

Seven GG cocks were mated with 20 GG hens, which produced 363 progeny. As expected, the mating of cocks and hens with GG genotypes resulted in progeny with the same normal four-toed foot phenotype.

In the present study, because the GT genotype was not detected in any of the symmetrical four-toed cocks, three TT cocks were simultaneously mated to seven TT homozygote hens and seven GT heterozygote hens, and character segregation was then observed. When TT cocks and TT hens were mated, only 30% (6/20) of the offspring showed the four-toed phenotype, whereas the other 70% (14/20) of individuals displayed five digits on one foot or both, with 10 chickens symmetrically five-toed. Similarly, when TT cocks were mated with GT hens, five-toed chickens were observed in the progeny flock at even larger numbers compared with the four-toed birds. The percentage of progeny with four toes on both feet was 13.2% (7/53), and the percentage with five toes on both feet and only one foot were 67.9% (36/53) and 18.9% (10/53), respectively.

#### Mating between symmetrically five-toed flocks

The mating experiment in the five-toed flocks was conducted in the following manner: eight TT cocks were mated with 20 TT hens; three of the eight TT cocks were simultaneously mated with six GT hens; and two of the GT cocks were mated to two GT hens separately. The resulting chicks showed almost all of the foot pattern types, suggesting the incompletely penetrant nature of digit number trait in Beijing You chickens.

Among the 310 offspring produced from the TT flocks, 287 chicks showed symmetrically five-toed patterns similar to their parents at a ratio of 92.58%. Additionally, two birds were characterized with six-toes on one foot. However, 5.16% (16/310) of individuals presented four digits on one foot and five digits on the other. Only five birds (1.61%) were bilaterally four-toed.

Seventy-two chicks were generated from the six GT hens sired with three TT cocks, among which 64 individuals appeared with five toes for both feet and five on one side. However, the remaining three chicks presented four toes on both feet. The gene penetrance of the five-toed trait was 95.83% (69/72), which was slightly lower than that observed from the TT×TT combination.

Two GT hens were inseminated by two GT cocks separately, and 19 offspring were generated. The number of chicks with four toes on both sides was 8, which was almost half of the total number of offspring. One chick had two extra digits on the left side, and the other ten chicks were all symmetrically five-toed. No unilaterally five-toed birds were observed in this group.

### Anatomical observations

**Fig 2:**
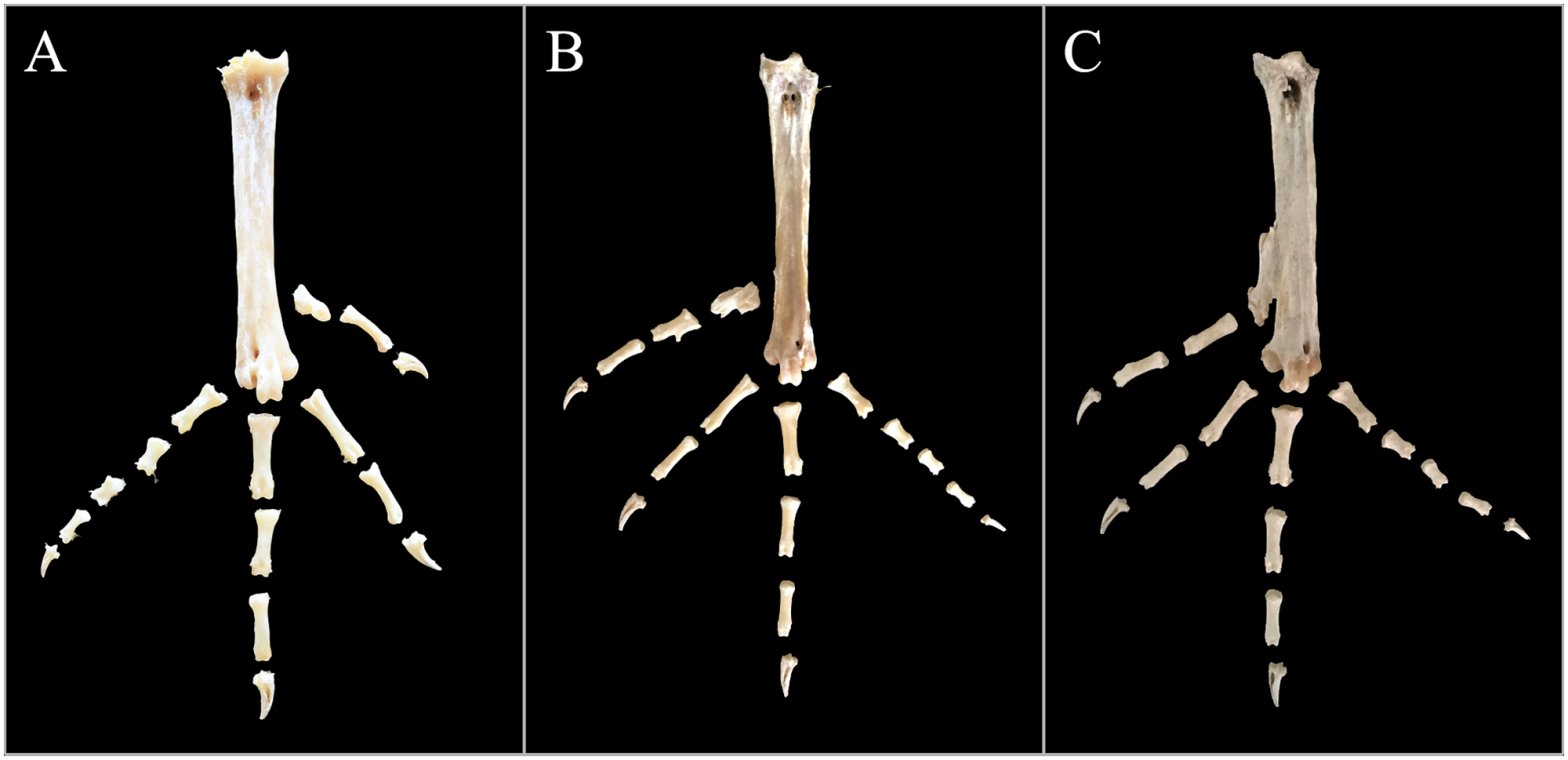
Typical four-toed foot bone arrangements in the Beijing You chicken population.

**Fig 3:**
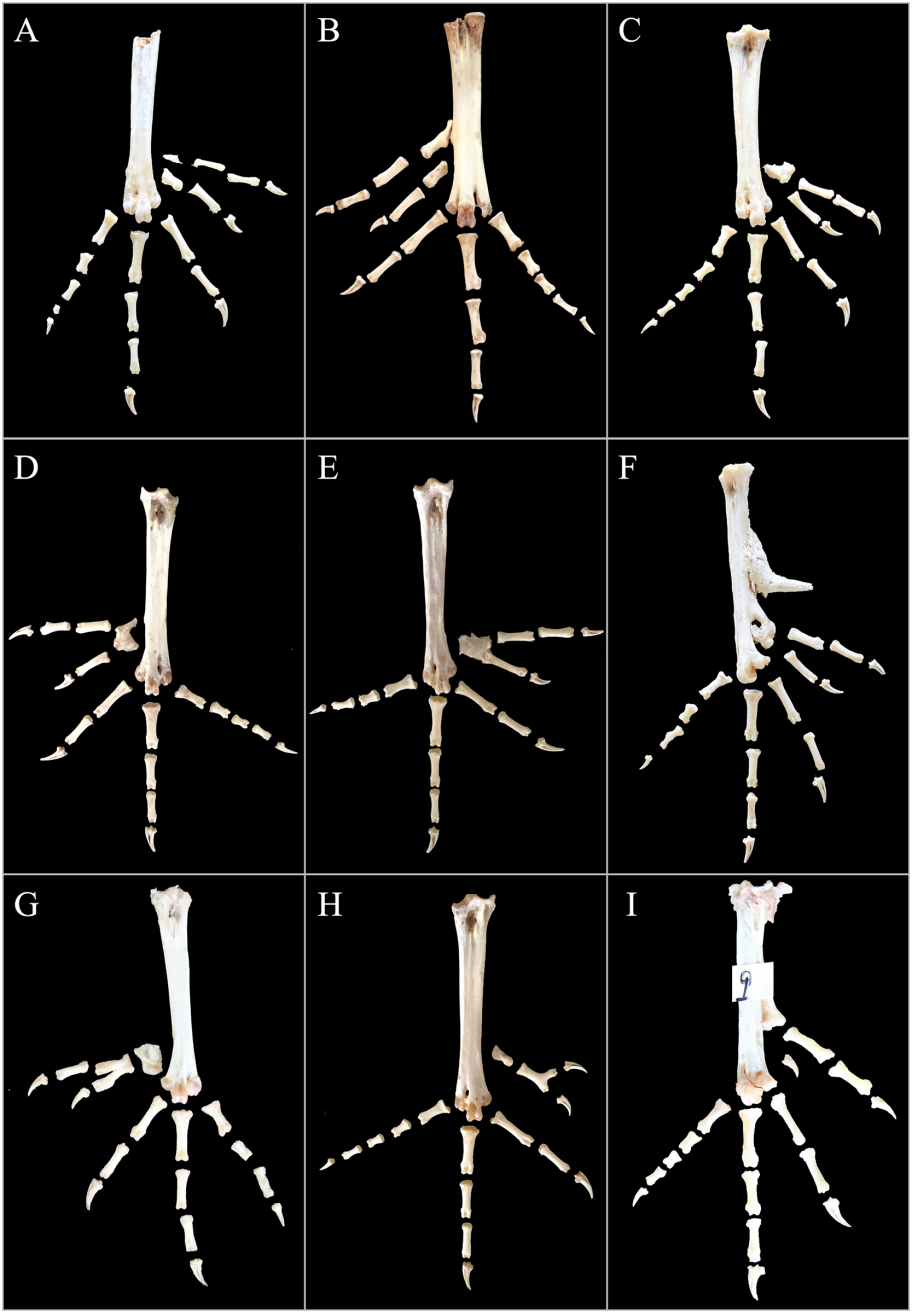
Typical five-toed foot bone arrangements in Beijing You chicken population. The image of the skeletal structure of the cock (F) shows a spur bone on the inner and medial side of the tibiotarsus.

**Fig 4:**
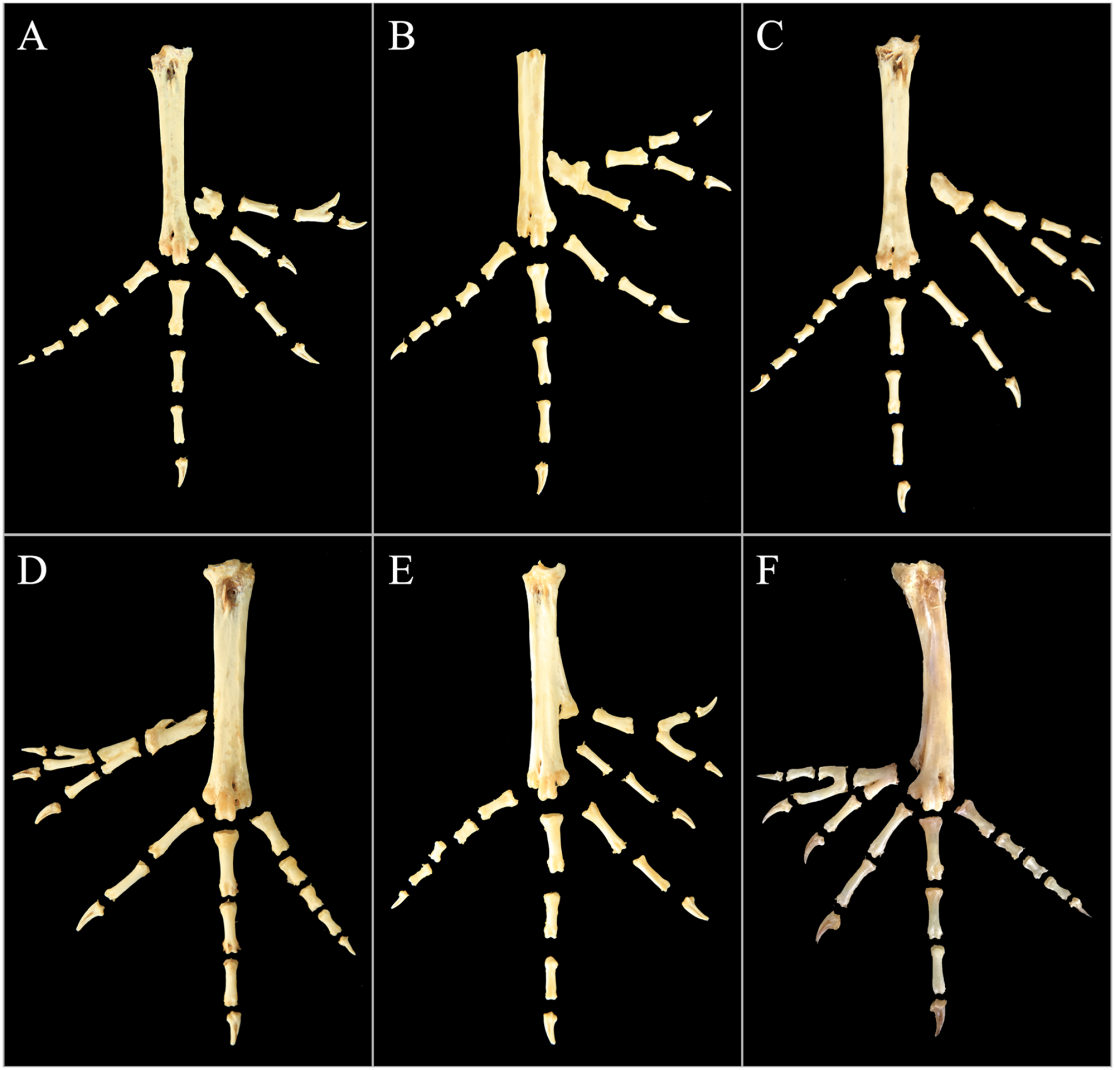
Typical six-toed foot bone arrangements in the Beijing You chicken population.

To examine the skeletal structures, a total of 116 chickens were slaughtered. Both feet of every bird were sampled, dissected and observed. The representative skeletal photos are illustrated in Figs 2–4.

#### Anatomy of four-toed feet

A total of 106 four-toed feet were collected, including 88 feet from 44 symmetrically four-toed chickens and 18 feet from unilaterally polydactylous birds, which included 13 birds with the 5/4 phenotype, four birds with the 4/5 phenotype and one 6/4 chicken. Only three relatively simple skeletal types were detected (Fig 2).

Coincidentally, all 27 GG birds were bilaterally four-toed, and their skeletal structures were exactly the same, with the first digit consisting of one metatarsal bone, one phalanx and one nail (Fig 2A). However, for the 10 four-toed birds with TT genotypes, two skeletal structures were observed. Eight out of 10 birds showed the bone structure illustrated in Fig 2B in which one more independent phalanx on digit one. The other two birds showed the skeletal type illustrated in Fig 2C in which the metatarsal bone was further fused with the tarsometatarsus, which is similar to the other three digits with metatarsals fusing with the distal tarsals to form a single tarsometatarsus bone. Typically, tiny furcations were observed on the metatarsal or the proximal phalanx bone from types B and C.

Among the seven GT hens, four hens had an additional phalanx symmetrically as observed in the skeletal pattern illustrated in Fig 2B, whereas the other three hens had the normal skeletal structure on both feet, which was similar to those from the GG genotype (Fig 2A). In addition, among the 18 four-toed feet from asymmetrically polydactylous chickens, 14 feet showed the same polyphalangy bone structure illustrated in Fig 2B, whereas the other four feet had normal four-toed bone structures, as shown in Fig 2A.

Briefly, the anatomy results revealed that only GG four-toed chickens represented actual four-toed individuals, which presented the digit conformation of 4-3-2-1. Chickens with the TT or GT genotype included two conditions, the normal four-toed flock and those with an additional phalanx on the first digit (4-3-2-2’ digit conformation), which were referred to as polyphalangy.

#### Anatomy of five-toed feet

In Beijing You chicken, the skeletal patterns of the two feet from the same polydactylous chicken were commonly non-identical. Hence, all bone arrangements of the 106 five-toed feet obtained from all 43 individuals with the 5/5 phenotype and 20 feet from bilaterally asymmetric phenotypic birds were categorized together.

Five-toed feet, which were only observed for birds with the TT or GT genotype, had several arrangements of skeletal structures (Fig 3). For a number of individuals, the bones of the accessory digits were completely different from those of the first digit (Fig 3A and 3B). The first digit was unaffected, while the accessory digit consisted of one small metatarsal bone, two phalanxes and a nail.

Most cases presented bone arrangement modes similar to those shown in pictures C-E (Fig 3), in which the metatarsal bone of the first digit and the additional digits were partially or completely fused and the distal bone ended in two articular surfaces, which corresponded to two digits. Moreover, in picture E, the metatarsal of the extra digit as well as the phalanx of digit one were not completely separated from the metatarsal bone, and the bone arrangement could be classified as the same type shown in photos C and D.

In addition, under certain conditions, the fused metatarsal was further conglutinated with the tarsometatarsus and generated a knot in the middle of the tarsometatarsus (Fig 3F). Moreover, in addition to the metatarsal bone, the phalanxes of the hallux and the adjacent extra digit were similarly fused together (Fig 3G). In certain cases, the metatarsal was regular but the phalanx bifurcated as shown in picture H, with two digits formed at the end, which were inconsistent with the fused digits. However, the incidence of such cases was low.

Compared with those of the normal four-toed feet (Fig 2A), the skeletal elements of digits two, three and four from the five-toed feet were unaffected, and even the bone composition of digit one remained normal, with one metatarsal bone, one phalanx and one nail, except for another supernumerary digit added to the outside of the first digit, which typically consisted of one small metatarsal bone, one or two phalanxes and a nail. Nevertheless, we observed one foot possessing an additional nail between the first digit and second digit (Fig 3I).

According to the composition and distribution of bones in the accessory digit, we categorized the 106 five-toed-feet bone constructions into six subtypes, and the numbers are provided in Table 4. The ratio of bone shape A/B which had hallux and the accessory digit completely separated was the highest, followed by that of C/D/E, with the metatarsal of the first and extra digit fused but apart from the tarsometatarsus. For subtype F the fused metatarsal was further joint with the tarsometatarsus and for that of G not only the metatarsal but also the proximal phalanx were fused, and therefore, we classified them separately. In fact, skeleton subtypes A/B, C/D/E, F and G belonged to the same type, with the extra digit appearing similar to digit two (4-3-2-1-2’ digit conformation). Subtype H presented another digit conformation 4-3-2-1-1’, with the extra digit having a similar skeletal form as digit one, although the proportion was rare (only approximately 4%). Subtype I was unique, with digit one displaying an extra phalanx and a superfluous nail between digits one and two, similar to polyphalangy. Additionally, the skeletal structures of 5/5 chickens were compared between the GG and GT genotypes; however, significant differences were not observed.

**Table 4:** Categories of five-toed feet with various bone arrangements.

Phenotype	Genotype	Bone type						Total
		A/B	C/D/E	F	G	H	I	
5/5	TT	30	25	2	4	-	1	62
	GT	10	8	1	3	2	-	24
5/4		6	4	-	2	1	-	13
4/5		1	1	1	-	1	-	4
6/5		1	1	-	1	-	-	3
Total		48	39	4	10	4	1	106

#### Anatomy of six-toed feet

In the present study, 12 chickens displaying the 6/- foot model were analyzed, with eight birds having six toes bilaterally, one bird having four toes on one side, and the other three birds possessing five toes on one side. The photos shown in Fig 4 exhibit the typical foot bone assembly models of six-toed feet. Similarly, in the six-toed feet, digits two, three and four were also unaffected. However, the structure and composition of the first digit varied.

In a small portion of the six-toed feet, the bone components of the hallux were unaffected (Fig 4A and 4B), although the metatarsal was fused with the components of the additional two digits. In photo A, the nail of the innermost digit conglutinated with the distal phalanges. These two bone conformations could be considered 4-3-2-1-2’-2’. Most chickens showed an extra phalanx in the first digit (Fig 4C–4F). For picture C-E, the skeleton pattern was 4-3-2’-2’-2’, with digit one and the two extra digits all having two phalanges. Typically, the hallux failed to split from the two additional digits, and in some instances, the unsplit metatarsals further adhered to the tarsometatarsus (E and F in Fig 4). In picture F of Fig 4, the number of bones was the maximum observed in the present study, and the additional two digits were “mirror images” of digits two and three (4-3-2-2’-2’-3’), with the hallux as the central axis.

## Discussion

Polydactyly is a phenotype observed in a number of domestic chicken breeds [3, 4, 27]. In the present study, the bone structures of polydactylous and normal Beijing You chickens were intensively and systematically studied at an anatomic level for the first time. We observed that bone arrangements was much more complex than phenotype appearances. Based on the appearances and the toes relative lengths, Warren [28] classified polydactylism of the feet into five major types and He et al. [2] further described another three subtypes. All these subtypes except for the type C were observed in our experimental population (S1(a)–S1(i) Fig). However, in the mass conserved Beijing You population we found this unique type (type C), characterized by two long digits split or unsplit in the inner side of the toe (S2 Fig).

In our study population, bone conformation 4-3-2-1-2’ was the most commonly observed digit type, which was also the most usual form in the Silkie chicken breed [29]. The appearances of this bone conformation correspond with the typical type B by Warren [28] and type A’ by He et al. [2]. However, various bone fusions or perhaps failures in separation were observed in the first digit and the extra digit (Fig 3). Type D and type B’, which possessed a short extra toe, usually exhibited the bone type of H in Fig 3, with the extra digit having a similar skeletal form as digit one, showing the digit conformation 4-3-2-1-1’.

Type E and G were two six-toed types described previously. However, for six-toed feet, whether the appearances (S1(j)–S1(l) Fig) or the bone arrangements (Fig 4) were more complicated than expected. The additional digits could have been produced from any metatarsal or phalange bone, with each displaying a distinct bone appearance, thus increasing the complexity of the skeletal structures. In addition to the usual polydactyly with one or more obvious extra digits, polyphalangy, categorized as type A was also observed as described by Warren [28]. Polyphalangy is a variant of polydactyly, the inner toe of which being relatively long than normal four-toed because of the existence of an extra phalanx.

In the present study, the ZRS in GGA2 was sequenced in Beijing You chickens, and no other mutations except for SNP rs80659072 were detected, suggesting that the ZRS sequence is highly conserved [30]. The association analysis showed the G/T polymorphism of rs80659072 was significantly associated with polydactyly in the mass Beijing You population (*P*<0.0001). Our results revealed that (1) all GG individuals were normal four-toed; (2) all polydactylous birds, including polyphalangy individuals, had one or more T alleles; and (3) seven GT or TT individuals, including 3 bilaterally four-toed birds and 4 asymmetrically polydactylous birds, carried the normal four digits on one or two sides.

Furthermore, a selective mating experiment demonstrated that normal four-toed GG flocks could never produce progeny with supernumerary digits. GG is a completely recessive congenital normal four-toed genotype. However, four-toed GT or TT individuals, whether with the normal four digits or a polyphalangy phenotype, generated more than 70% polydactylous offspring on one or both feet. Moreover, mating among symmetrically five-toed TT flocks produced 2% of chickens with bilaterally four digits. Although most were polydactylous with a longer inner appearance, only a few birds possessed the normal four digits, which confirmed the dominant and incompletely penetrant hereditary pattern of the polydactyly trait in chickens [3]. Therefore, although a few birds with the normal four-toed phenotype were observed, SNP rs80659072 can still be used as a genetic marker for the selection of polydactyly in Beijing You chickens.

Noticeably, these findings suggest that the rs80659072 genetic variant in the chicken ZRS region was required but not sufficient to produce polydactylous conditions in Beijing You chickens, which is somewhat inconsistent with the findings of previous reports [2, 4]. This discrepancy may be due to differences in the flocks [28] and variations in the genetic backgrounds. Moreover, additional genetic differences could prevent the same substitution from having the same functional consequences in more distant ancestral backgrounds [31].

For polydactyly in chickens, the well-known SNP in the ZRS region was not the sole causative agent. Various genetic mechanisms underlying polydactyly phenotypes have been reported. Dorshorst et al. [3] suggested that although polydactyly appeared as a single gene trait in different chicken breeds, more than one allele or locus might control polydactyly in chickens and rs80659072 was a candidate for one of at least two causal mutations. Zhang et al. [4] documented that polydactyly phenotypes in European and Chinese chickens were caused by two closely located but independent mutation events. Robb et al. [30] conducted an inheritance analysis of the phenotypic variation in a congenic polydactyly chicken line and suggested that additional modifying genetic elements located outside of the ZRS could impact the preaxial polydactyly phenotype. Besides, in Dorking, Bouldin and Harfe [32] disclosed that preaxial polydactyly was initiated by the up-regulation of *fibroblast growth factor 4* (*Fgf4*) expression instead of *Shh*.

In the mating experiment, a very interesting observation was that the mating of TTxTT animals resulted in different proportions of four-toed offspring depending on the parent’s phenotype, with 30% (6/20) for four-toed TT-animals and 1.6% (5/310) for five-toed ones, respectively. This suggests a role of the genetic background. In addition to the dominant locus, other genetic elements may affect the penetrance of chicken polydactylism. To gain understanding of the additional genetic elements, a genome-wide association study (GWAS) incorporating the ZRS mutation genotype as a fixed effect would be a useful and powerful tool [33].

Polydactyly occurs during the anterior-posterior patterning of the developing limb bud, where the spatiotemporal regulation is very complex. The phenotypic variability might be controlled by either genetic or epigenetic mechanisms or both [30]. In addition, incubation temperatures also influenced the expression of a polydactylous phenotype in many chicken breeds [28, 30, 34]. Hence, further studies are recommended to unravel the mechanisms behind the polydactyly phenotype in Beijing You chickens.

## Conclusions

The SNP rs80659072 in the chicken ZRS sequence played an obvious and crucial role in the production of polydactyly phenotypes in Beijing You chickens. This polymorphism could be used as a molecular marker of polydactyly in Beijing You chickens. However, a number of flocks showed the normal four digits albeit the presence of the mutant (T) allele. Therefore, further in-depth investigations are required to elucidate the complex morphological phenotype of chicken polydactyly.

## Supporting information

S1 Fig Different foot phenotypes observed in Beijing You chickens. (a)-(i): Foot subtypes described by Warren [28] and He et al. [2]. The red letters in the lower-right corner correspond to different subtypes. (j)-(l): Other six-toed polydactylous foot discovered in this study.
(TIF)

S2 Fig Phenotype appearances and bone arrangements of type C classified by Warren [28]. (a) and (b): polydactylous foot with the inner side carrying two long digits split (a) or unsplit (b). (c) and (d): The skeleton structure photos corresponding to (a) and (b), respectively. The two inner digits are relatively long because of the existence of an extra phalanx.
(TIF)
