# Supplementary figures and images for: Association of SNP rs80659072 in the ZRS with polydactyly in Beijing You chickens
Source: PLoS One. 2017 Oct 9;12(10):e0185953. doi: 10.1371/journal.pone.0185953 (PMC5633194; doi:10.1371/journal.pone.0185953)

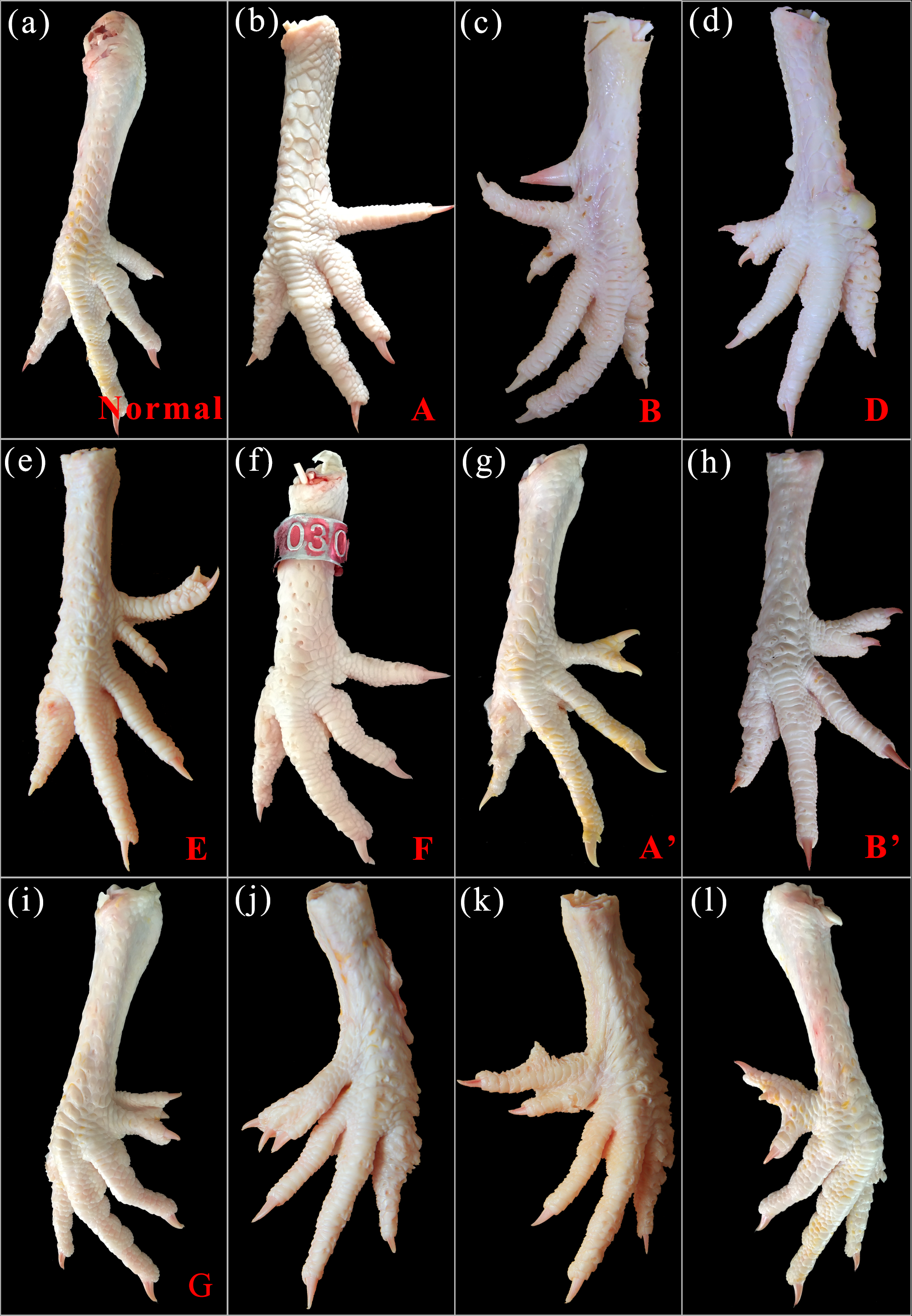

Supplement: S1 Fig — (a)-(i): Foot subtypes described by Warren [28] and He et al. [2]. The red letters in the lower-right corner correspond to different subtypes. (j)-(l): Other six-toed polydactylous foot discovered in this study. (TIF) [file pone.0185953.s001.tif]

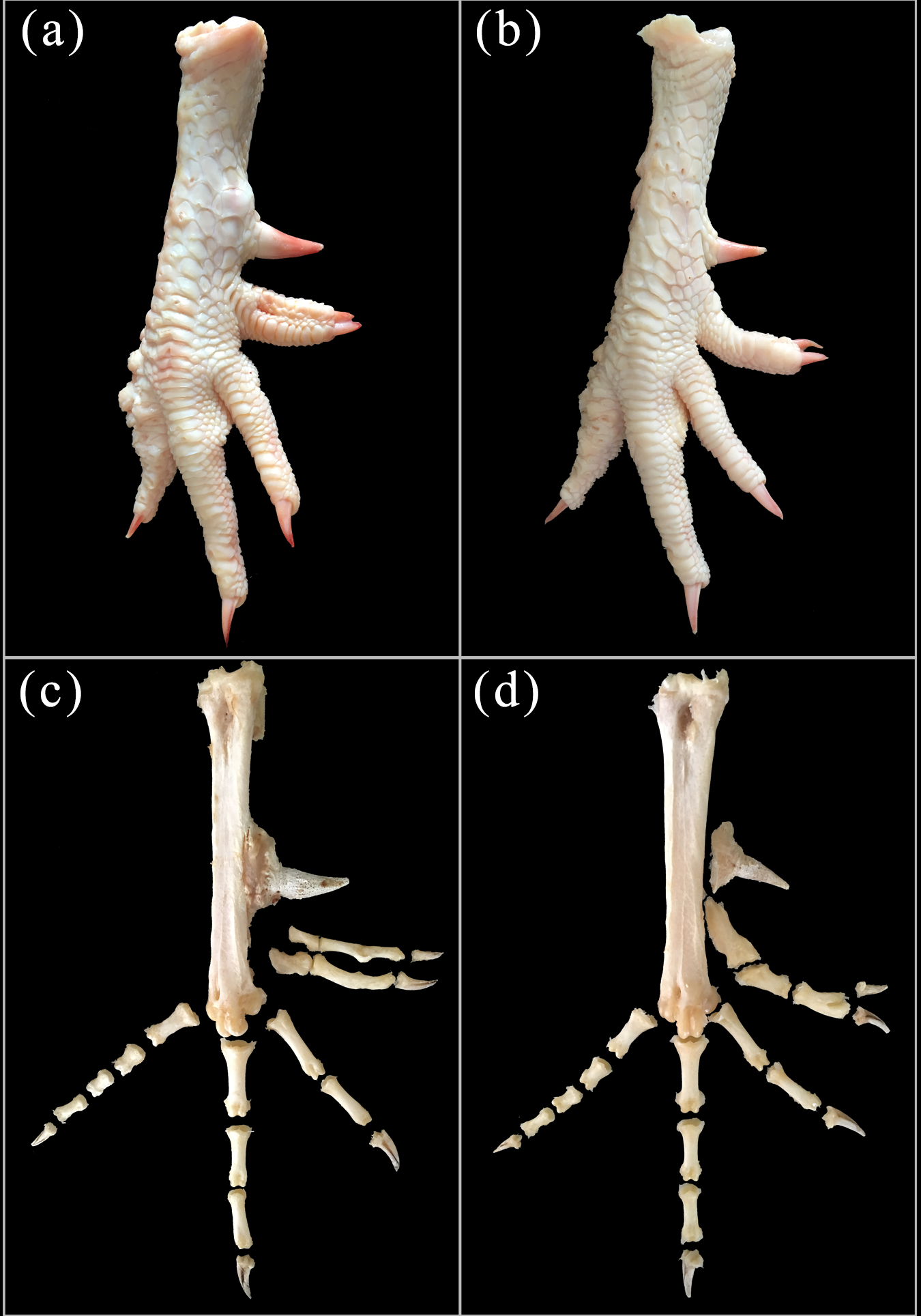

Supplement: S2 Fig — (a) and (b): polydactylous foot with the inner side carrying two long digits split (a) or unsplit (b). (c) and (d): The skeleton structure photos corresponding to (a) and (b), respectively. The two inner digits are relatively long because of the existence of an extra phalanx. (TIF) [file pone.0185953.s002.tif]
